# Effects of a brown rice-derived supplement on physical, cognitive and mental health among adults and the role of the gut microbiota: study protocol for a longitudinal, double-blind, randomized, placebo-controlled trial

**DOI:** 10.1186/s12906-026-05384-5

**Published:** 2026-04-25

**Authors:** Michio Takahashi, Keisuke Kokubun, Mayuko Yoda, Shinpei Kawaoka, Taizen Nakase, Ohara Tadashi, Yasuyuki Taki

**Affiliations:** 1 Smart-Aging Research Center, Tohoku University, 4-1 Seiryo-Machi, Aoba-Ku, Sendai, Miyagi 980-8575 Japan; 2 Department of Brain Healthcare Business Ecosystem, Graduate School of Management, Kyoto University, Kyoto, Japan; 3 Department of Integrative Bioanalytics, Institute of Development, Aging and Cancer, Miyagi, Japan; 4 Department of Aging Research and Geriatric Medicine, Institute of Development, Aging and Cancer, Tohoku University, Miyagi, Japan; 5 Department of Gastroenterology, Akirudai Hospital, Tokyo, Japan; 6 Brainbiome, Inc., Tokyo, Japan

**Keywords:** Brown rice, Immune system, Gut microbiota, Cognition, Mental health, Gut–brain axis

## Abstract

**Background:**

The consumption of brown rice, which contains bran and germ and is rich in fiber, micronutrients, and phytochemicals, can contribute to good physical, cognitive, and mental health. However, these health benefits and the relevant mechanisms have not been fully elucidated, especially in human subjects. Therefore, we will conduct a randomized controlled trial to examine the effects of a brown rice-derived supplement on various aspects of health and to elucidate the underlying mechanisms.

**Methods:**

This study will be a prospective, longitudinal, double-blind, randomized, placebo-controlled trial. Eighty healthy adults aged 20 to 64 years will receive 3.5 g/day of a brown rice-derived supplement or placebo for 12 weeks. We will assess immune-related and metabolic markers using transcriptome and metabolome analyses of blood samples, respectively, and evaluate the gut microbiota composition using stool samples, including 16S rRNA-based analysis, with short-chain fatty acids also measured. Cognitive function, constipation, sleep, mood state, and health-related quality of life will also be assessed using validated assessments at baseline and at the endpoint. Between-group comparisons will be conducted using appropriate statistical methods according to the distribution and characteristics of the data.

**Discussion:**

This study is the first to examine the effects of a brown rice-derived supplement on various aspects of health, including immune, cognitive, physical and mental health. We hypothesize that changes in the variation in the gut microbiota play a key role in the mechanisms underlying these health effects; therefore, we will investigate how changes in the gut microbiota caused by the consumption of the brown rice-derived supplement affect health status. We hope that the information derived from this study will be useful for promoting the use of brown rice-derived supplements as a healthy food and improving public health.

**Trial registration:**

University Hospital Medical Information Network Clinical Trials Registry (UMIN-CTR), UMIN000049330. Registered on 8 November 2022. https://rctportal.niph.go.jp/en/detail?trial_id=UMIN000049330

**Table:** 

Title {1}	Effects of a brown rice-derived supplement on physical, cognitive and mental health among adults and the role of the gut microbiota: Study protocol for a longitudinal, double-blind, randomized, placebo-controlled trial
Trial registration {2a and 2b}.	University Hospital Medical Information Network Clinical Trials Registry (UMIN-CTR), UMIN000049330.
Protocol version {3}	Protocol version 4, 3 Feb. 2023
Funding {4}	The Isyoku Dougen Syouyaku Kenkyu Foundation
Author details {5a}	Michio Takahashi, Keisuke Kokubun, Mayuko Yoda, Shinpei Kawaoka, Taizen Nakase, Ohara Tadashi, Yasuyuki Taki
Name and contact information for the trial sponsor {5b}	Yasuyuki Taki 4–1 Seiryo-Machi, Aoba-ku, Sendai, 980–8575, Japan
Role of sponsor {5c}	The Isyoku Dougen Syouyaku Kenkyu Foundation has not had and will not have any impact on the stages of the trial, including data collection, analysis and interpretation of data and the publication process.

## Introduction

### Background and rationale {6a}

Considerable attention has been given to the consumption of whole grains to improve health conditions. In the case of rice, accumulating evidence has suggested that the consumption of brown rice, which contains bran and germ and is rich in fiber, micronutrients, and phytochemicals [1, 2], can improve physical health (e.g., immune function, sleep, and obesity control) [3–7], cognitive health (e.g., general cognitive function and executive function), and mental health (e.g., depression and anxiety) [2, 8–10]. However, these health benefits have not been fully elucidated, especially in human subjects. For example, the positive effects of γ-oryzanol—a mixture of lipids that are abundant in brown rice—on the immune system have been demonstrated in animals through in vivo and in vitro studies but not in humans [11, 12]. Moreover, the mechanism underlying the health benefits of brown rice consumption remains unclear. Therefore, it is important to clarify the health benefits of brown rice and the mechanism underlying these beneficial effects to promote the consumption of brown rice from a public health perspective.

Regarding the mechanism underlying the beneficial effects of brown rice consumption, we assume that one of the important factors associated with these positive effects on health may be the change in intestinal microbial ecology caused by the consumption of brown rice. Whole grains balance the gut microbial ecology by increasing microbial diversity and inducing compositional alterations that benefit the host [3, 4]. Recent studies have reported that consuming brown rice positively affects the intestinal environment and improves physical health. A human study reported that consumption of a beverage made from brown rice for 4 weeks led to increases in the abundance of beneficial species in the intestine, which were associated with reduced inflammation and increased short-chain fatty acid production in patients with metabolic syndrome [13]. These results indicate that the consumption of brown rice exerts a positive effect on the immune system by changing the composition of the gut microbiota. The reason for this effect may be that brown rice is rich in dietary fiber, which produces short-chain fatty acids in the intestines, thereby positively affecting the immune system. Another study reported that consuming brown rice decreases the serum levels of inflammatory factors such as IL-6 and IL-8 by balancing peripheral blood mononuclear cells (PBMCs) and increasing short-chain fatty acids (SCFAs) in the gut [14]. However, this study did not capture the whole effect on the immune system because it specifically examined cytokines. Furthermore, the abovementioned studies were limited to individuals with metabolic symptoms and thus did not report how brown rice functions in the immune system in the general adult population, including individuals without health problems. Previous studies have suggested that certain dietary habits have a positive effect on the immune system and maintain a good health status in healthy adults [15–17]. Additionally, knowledge regarding the various health-related effects of brown rice, as well as knowledge of the immune system, has been very limited in humans. For example, brown rice may modulate the gut microbiota and thus affect physical and cognitive health [18–21]. Regarding obesity, animal studies have suggested that the intestinal microbiota affects obesity by prompting reward signals in the dopaminergic system [22, 23]. However, these mechanisms have not been elucidated in humans. Additionally, a healthy gut environment that balances microbiota diversity contributes to good mental health, but no previous study has investigated how brown rice consumption can balance the gut microbiome environment and how this phenomenon affects mental health [24].

Furthermore, despite its overall health benefits, brown rice is not widely accepted for consumption in daily meals. One of the problems with the frequent intake of brown rice is its hard texture even after cooking [25, 26]. This hard texture occurs because the wax in the outermost layer of brown rice grains inhibits water absorption [27]. To address this issue, we focus on the use of a brown rice-derived supplement as an alternative to eating brown rice. The brown rice-derived supplement that we will use in the present study includes the key nutrient-rich parts of brown rice, extracted and made into powder, thus making it easy to consume the active ingredients contained in brown rice for health purposes. Among these constituents is γ-oryzanol, which is a characteristic component of brown rice and has been suggested to exert beneficial effects on metabolic and inflammatory processes in experimental studies [11, 12, 18]. In addition, dietary fiber may be relevant to the present trial because it can influence the gut microbiota and related immune regulation [13, 14]. Therefore, this supplement may influence health through multiple biological pathways, especially immune function and gut microbiota-associated mechanisms. However, the effects of brown rice-derived supplements on physical, cognitive, and mental health have not been examined. When the health-promoting efficacy of food products is being examined, distinct products need to be examined separately if they have substantially different amounts and ratios of components, even if they are derived from the same food; there are likely synergistic interactions between components, and differences in composition can strengthen or weaken the effects of consuming specific products [28, 29].

### Objectives {7}

The objective of this trial is to perform a longitudinal investigation of the influence of consuming a brown rice-derived supplement for 12 weeks on multiple health-related outcomes in healthy adults. In particular, we will assess measures related to immune function, gut microbiota, cognitive function, mental and physical health, sleep, constipation, and health-related quality of life (HRQOL). We hypothesize that participants receiving the brown rice-derived supplement will show favorable changes in these outcomes compared with those receiving placebo and that changes in the gut microbiota may partly underlie these effects. Therefore, we will also examine the relationships between changes in the gut microbiota and various health aspects derived from the consumption of the brown rice-derived supplement.

### Trial design {8}

**Fig. 1:**
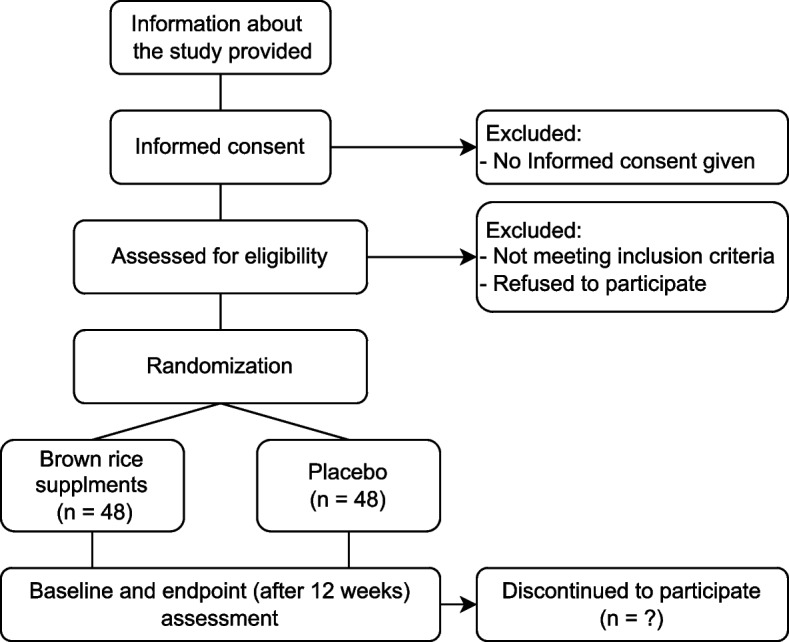
Overview of the planned trial design

This study will be a longitudinal, double-blind, randomized, placebo-controlled prospective intervention trial with a parallel group of healthy adults (see Fig. 1 for an overview of the planned trial design). We designed this study to explore the effects of brown rice-derived supplementation on various health outcomes, including the immune system, cognitive function, gut microbiota, constipation, sleep, and HRQOL. Participants will be randomly assigned to the brown rice-derived supplement group or the placebo group at a ratio of 1:1. This protocol is in accordance with the Standard Protocol Items: Recommendations for Interventional Trials checklist [30]. This study protocol was approved by the Ethics Committee of Tohoku University Graduate School of Medicine (IRB# 2022–1–845) and preregistered in a clinical trial registry (UMIN-CTR ID: UMIN000049330). The committee has peer-reviewed the progress of the study, independent of our research team.

## Methods: participants, interventions and outcomes

### Study setting {9}

The study will be conducted at the Smart-Aging Research Center of Tohoku University (Japan), and all the data will be collected at the center.

### Eligibility criteria {10}

The inclusion and exclusion criteria for participation in this trial are listed in Table 1.

**Table 1:** Inclusion and exclusion criteria

Inclusion criteria	Exclusion criteria
• Adults aged 20 to 64 years at registration • Having a willingness to consume experimental products daily for 12 weeks at the entry • Informed consent can be obtained	• Estimated IQ < 80 • Severe visual and hearing impairment • Having habits of consuming brown rice and related supplements in the past year • Pregnancy or breastfeeding • Currently participating in other clinical trials • Following disease with a severe level – Endocrine and metabolism disease – Heart disease – Digestive system disease – Severe allergic disease – Dementia and neurological disease – Psychiatric disorder • Other reasons for difficulties in carrying out the research

### Who will take informed consent? {26a}

The research staff will obtain informed consent from potential participants who have been deemed preliminarily eligible. After the staff has determined eligibility and thoroughly explained the details of the study, potential participants will have the opportunity to ask questions. If participants are willing to participate in the study, they will sign the informed consent document.

## Interventions

### Explanation for the choice of comparators {6b}

We will use maltodextrin colored with edible food dye to resemble the brown rice-derived supplement used in this study. The reason for the use of maltodextrin is that it is similar in texture and taste to the brown rice-derived supplement.

### Intervention description {11a}

The participants who are assigned to the brown rice-derived supplement group will receive 3.5 g of the brown rice-derive supplement per day orally (“Kinme Mai Ekisu” from Toyo Rice Corp., Wakayama, Japan) for 12 weeks. The brown rice-derived supplement includes nutrient-rich parts of brown rice that have been extracted and made into powder. It contains several bioactive components that may be relevant to the present trial, particularly γ-oryzanol and dietary fiber. The participants who are assigned to the placebo group will receive 3.5 g of placebo per day orally. The placebo will have a similar appearance to the brown rice-derived supplement. The supplement and placebo will each be packaged in a 3.5 g stick, and participants will be instructed to consume one stick per day at any time of day they choose. Both the supplement and placebo will include only products that have been confirmed to be safe in Japan and have been marketed as food products. The supplement and placebo are not expected to cause serious adverse effects. The nutritional information of the brown rice-based supplement and placebo is shown in Table 2.

**Table 2:** Nutrient composition of brown rice-derived supplement and placebo

	Brown rice-derived supplement		Placebo	
	Per 100 g	Per package (3.5 g)	Per 100 g	Per package (3.5 g)
Protein	17.3 g	0.61 g	< 0.1 g	< 0.01 g
Crude fat	26.4 g	0.92 g	< 0.1 g	< 0.01 g
Carbohydrates	40.3 g	1.41 g	95.3 g	3.34 g
Sugar	23.0 g	0.81 g	94.3 g	3.30 g
Dietary Fiber	17.3 g	0.61 g	1.0 g	0.04 g
Calories	433.0 kcal	15.16 kcal	379.0 kcal	13.27 kcal
Sodium	14.1 mg	0.49 mg	86.4 mg	3.02 mg
Phosphorus	2710.0 mg	94.85 mg	1.1 mg	0.04 mg
Iron	9.83 mg	0.34 mg	N/A	N/A
Calcium	58.1 mg	2.03 mg	N/A	N/A
Potassium	2020.0 mg	70.70 mg	N/A	N/A
Magnesium	1130.0 mg	39.55 mg	0.2 mg	0.01 mg
Copper	0.7 mg	0.02 mg	N/A	N/A
Zinc	8.8 mg	0.31 mg	N/A	N/A
Manganese	15.4 mg	0.54 mg	N/A	N/A
Thiamine	4.6 mg	0.16 mg	N/A	N/A
Vitamin B6	3.6 mg	0.13 mg	N/A	N/A
Folic acid	97.0 μg	3.40 μg	N/A	N/A
Niacin	47.4 mg	1.66 mg	N/A	N/A
α-Tocopherol	19.5 mg	0.68 mg	N/A	N/A
β-Tocopherol	0.8 mg	0.03 mg	N/A	N/A
γ-Tocopherol	2.3 mg	0.08 mg	N/A	N/A
δ-Tocopherol	0.1 mg	0.004 mg	N/A	N/A
GABA	214.0 mg	7.49 mg	N/A	N/A
Ferulic acid	0.3 g	0.01 g	N/A	N/A
γ-oryzanol	200.0 mg	7.00 mg	N/A	N/A
Lipopolysaccharide	3440.0 μg	120.40 μg	0.6 μg	0.02 μg

*GABA* Gamma-aminobutyric acid, *N/A* Not available

### Criteria for discontinuing or modifying allocated interventions {11b}

We do not expect serious adverse effects to occur after consumption of the brown rice-based supplement or the placebo. However, if a participant wishes to discontinue the consumption of experimental products due to poor physical condition experienced during the intervention period, the intervention will be stopped for that individual. In the event of an allergic reaction to the experimental products, the intervention will be stopped immediately for the affected individual. The decision to discontinue consumption will be left to the individual participants, and any participant who chooses to discontinue the intervention will be asked to explain the reason. In this study, no modifications will be made regarding the allocated interventions or the dose of the experimental products.

### Strategies to improve adherence to interventions {11c}

During the intervention period, participants will complete a checklist that asks about the timing of consumption (morning, noon, or night) every day. In addition to the checklist, we will ask participants to fill out a web report system that was developed using Google Forms (Google LLC, CA, US) to report when they consumed the experimental product. The web report system will enable us to sequentially determine whether each participant has consumed the experimental product and to send reminders to participants if they have not consumed the stick by a certain time of day (ex. 19:00).

### Relevant concomitant care permitted or prohibited during the trial {11d}

We will ask participants to not intentionally change their eating habits. Participants will be allowed to take other supplements or medications, but they will be asked to report any new supplements or medications they take during the intervention period. In addition, we will ask participants to report their medical history, if any, during the intervention period.

### Provisions for posttrial care {30}

Severe adverse events, including unexpected allergic reactions resulting from the consumption of the brown rice-derived supplement or placebo, will be covered by clinical research insurance if the adverse effects result from the consumption of the supplement or placebo. Other potential physical harm will be covered by public insurance. There will be no posttrial care.

### Outcomes {12}

Previous studies have suggested that brown rice has various effects on human health, and we will present evidence concerning these effects and the underlying mechanisms by using a brown rice-derived supplement in this study. Therefore, the overall condition of the immune system, metabolism, constipation, sleep, cognitive function, mental health, and gut microbiota community composition will be co-primary outcomes for comprehensively examining various health aspects. Blood samples will be collected to analyze immune function and metabolism via transcriptome and metabolome analysis, respectively, and stool samples will be collected to evaluate diversity in the gut microbiota.

In addition, we will collect information on the factors that may influence the relationships between consumption of the brown rice-derived supplement and primary outcomes. Various lifestyles could influence the relationships between the consumption of the brown rice-derived supplement and broad health conditions. In particular, daily eating habits and physical activity may strongly impact these relationships. Therefore, we use the brief-type self-administered diet history questionnaire (BDHQ) and a simple questionnaire to evaluate total and domain-specific physical activity [31, 32]. In addition, we will collect data on the overall intelligence quotient (IQ) to control for the influence of IQ on the performance of cognitive tests [33].

Since this study aims to determine the effects of brown rice-derived supplement consumption on various health aspects, all sample collection and measurements, except for daily health, will be performed at two time points (baseline and endpoint), and we will compare the changes in these variables between and within groups. Daily health during the intervention period will be compared between groups. We assume that these variables will change in a positive direction if the brown rice-derived supplement is effective at improving human health.

### Participant timeline {13}

**Fig. 2:**
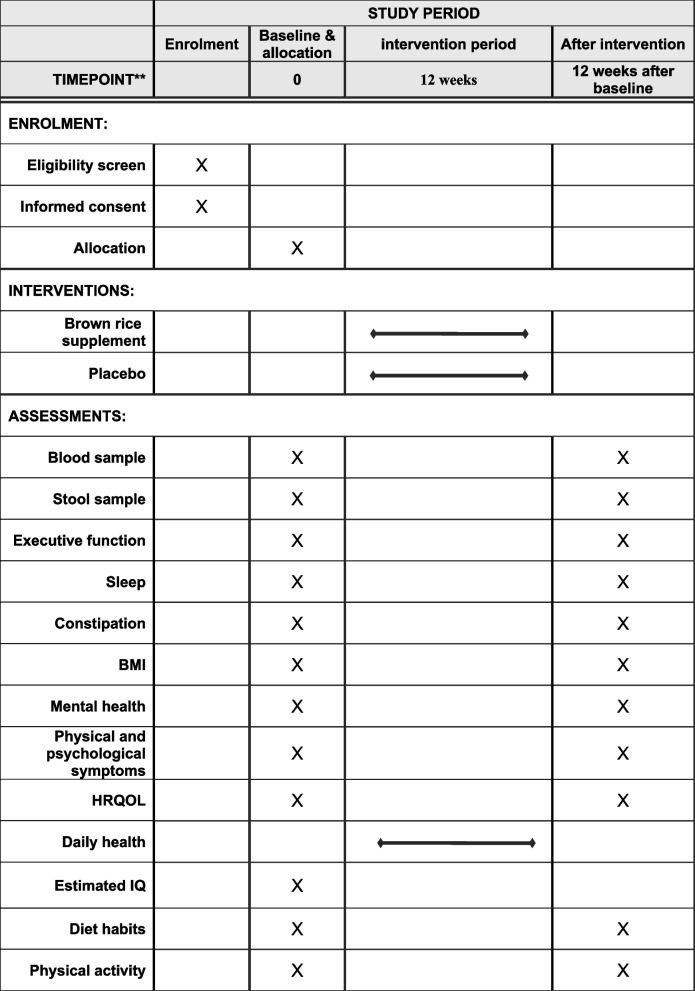
Schedule of enrollment, intervention, and assessments for participants

A schematic diagram of the time schedule for the participants is shown in Fig. 2.

### Sample size {14}

This is the first study to simultaneously examine the effects of a brown rice-derived supplement on physical, cognitive and mental health; therefore, determining a uniquely valid sample size is not possible. Instead, we determined the sample size for this study (i.e., 40 participants in each group) by referencing an RCT that examined the effect of processed brown rice products on health, including the gut microbiota [13]. We will recruit 96 participants to allow for a potential 20% dropout rate.

### Recruitment {15}

We will recruit all participants by posting an advertisement in a community information magazine that is freely distributed in Sendai City and surrounding cities in Miyagi Prefecture, Japan. Individuals who are willing to participate in the trial will be asked to access the website with detailed information via a QR code. Cash (7000 yen, if all protocols are completed) will be offered as remuneration for all participants. Recruitment will be conducted in three phases, with approximately 32 participants recruited per phase.

### Sequence generation {16a}

Participants will be randomly divided into a brown rice-derived supplement group and a placebo group at a 1:1 ratio by computer-assisted random number generation. To maintain double-blind conditions, neither the examiner nor the participants will have any information regarding which group they are allocated to during the study.

### Concealment mechanism {16b}

The information on the allocation of participants will be kept in a tightly sealed envelope and locked in a locked cabinet that is inaccessible to the examiner. The envelopes will be checked weekly by a laboratory staff member who will not participate in the trial and who has a desk in a separate room from the examiner. Allocation concealment will be guaranteed by the above procedures.

### Implementation {16c}

The participants will be allocated to groups by staff members who will not otherwise participate in the examination. Other technical staff in the laboratory will prepare the respective interventions and put them in the bags that will be given to participants on the basis of allocation numbers, but the numbers will not include information about the supplement or placebo.

## Assignment of interventions: blinding

### Who will be blinded {17a}

All involved parties, including participants and research staff members responsible for the allocation and assessment of outcomes, will be blinded, as the supplement and placebo will arrive having already been packaged, with only the identifying numbers printed on the products.

### Procedure for unblinding if needed {17b}

If severe adverse effects or allergic reactions occur, the consumption of the brown rice-derived supplement or placebo will immediately be stopped. If the principal investigator judges that knowledge of allocation might be beneficial for the participant’s safety, the sealed envelopes containing the table regarding assignment will be opened, and the individual assignment of the participant will be revealed to report severe adverse effects and allergic reactions by the research staff managing the table. After such reports are provided, the table will be strictly resealed and maintained.

## Data collection and management

### Plans for assessment and collection of outcomes {18a}

#### Immune function

PBMCs will be collected using BD Vacutainer® CPT™ Mononuclear Cell Preparation Tubes with sodium citrate (BD Bioscience, USA) according to the manufacturer’s instructions. Then, total RNA will be extracted from PBMCs using an RNeasy Mini Kit with an RNase-Free DNase Set (Qiagen, Venlo, Nederland). RNA-seq libraries will be generated using the NEBNext Globin & rRNA Depletion Kit and the NEBNext UltraII Directional RNA Library Prep Kit (New England Biolabs, MA, USA) according to the manufacturer’s instructions. Sequencing experiments will be performed with a NextSeq 500 (Illumina; High Output Kit v2.5, 75 cycles). The obtained reads will be mapped to the human genome grch38 and processed using fastp (removing reads with < Q30), HISAT2, SAMtools, and featureCounts [34–37]. The obtained TPM scores will be subjected to identification of differentially expressed genes (DEGs) [38], gene ontology analysis [39], estimation of immune cell contents [40], and weighted gene coexpression network analysis [41]. The data will be represented according to the examples described in our previous publications [42–46].

#### Metabolism

Plasma samples will be collected using BD Vacutainer® CPT™ Mononuclear Cell Preparation Tube Sodium Citrate (BD Bioscience, USA) in accordance with the manufacturer’s instructions. Metabolites will be extracted from plasma using Bligh and Dyer’s method with some modifications [47]. Hydrophilic metabolite analysis will be performed using LC with a Discovery HS F5 column coupled with an LCMS-8060NX triple quadrupole mass spectrometer (Shimadzu, Kyoto, Japan) in multiple reaction monitoring (MRM) mode (LC/MS/MS in MRM mode) as described previously [48]. The analytical platform for hydrophilic metabolite analysis will be controlled using LabSolutions (version 5.80; Shimadzu, Kyoto, Japan). The quantitative content of the hydrophilic metabolites will be calculated using the peak area relative to the internal standard (IS). The obtained datasets will be analyzed using the KEGG database [49], pipelines for identifying differentially expressed metabolites, and weighted gene coexpression network analysis. Examples of data representation have been described in our previous publications [43, 46, 50].

#### Gut microbiota

Stool samples will be collected by each participant at home using a stool collection kit containing a preservation solution one week prior to one day before the collection of blood and cognitive function data; we will receive the stool samples from participants on the day of the data collection. According to the manufacturer and a previous validation study of the preservative solution, fecal microbiota profiles can be stably maintained at room temperature for approximately one month [51]. After the stool samples are received from the participants, these samples will be refrigerated at ≤ 4 °C until shipment to the analytical laboratory (TechnoSuruga Laboratory Co., Ltd., Shizuoka, Japan). The stool samples preserved with the kit will be sent to the analysis institution within 4 weeks of collection to conduct 16S rRNA analysis and measure short-chain fatty acids (SCFAs). To determine the bacterial composition of the stool samples, 16S rRNA amplicon sequencing will be performed [52, 53]. This method provides an overview of the community composition of a bacterial community and reveals differences between groups and changes within groups. In the analysis, DNA will be isolated from stored stool samples and amplified by PCR, and sequence reads will be determined using the Illumina MiSeq system. The sequence reads will be imported into Quantitative Insights into Microbial Ecology version 2 (QIIME2) and then analyzed for bacterial identification and diversity [54, 55]. The principal gut microbiota outcomes in this study will be alpha diversity and beta diversity. Alpha diversity will be assessed using indices such as the Shannon index and observed features, whereas beta diversity will be evaluated using distance metrics such as Bray–Curtis dissimilarity and UniFrac distances. In addition, SCFAs in stool samples will be measured by high-performance liquid chromatography using a post-column reaction with a detector, two tandem columns, and a guard column.

#### Cognitive function

We will use neuropsychological tests to evaluate the overall domains of executive function, working memory, inhibitory control, and cognitive flexibility [56]. The stop signal task of the Cambridge Neuropsychological Test Automated Battery (CANTAB) (Cambridge Cognition, Cambridge Ltd, UK) will be used to evaluate response inhibition. In this task, participants are required to respond to an arrow stimulus by selecting one of two options, depending on the direction in which the arrow points. If an audio signal (a beep) is presented with an arrow stimulus, participants must withhold making that response and not select an option. The spatial span task of the CANTAB is used to evaluate visuospatial working memory. In the test, white squares are shown on the screen, some of which briefly change color in variable sequence; then, the participant is required to select the boxes that changed color in the same order that they were displayed on the screen (for the forward test) or in the reverse order (for the backward test). The number of boxes in the sequence increases from two to nine. Cognitive flexibility will be assessed with the Wisconsin Card Sorting Test (WCST) [57, 58]. In the test, participants are required to sort a series of cards according to different rules and alter their strategy when the rules change unexpectedly. In this study, we will use the computerized version of the WCST: Computer version 4 (PAR. Inc., Florida, US).

#### Sleep

We will assess nighttime sleep quality using a self-rated questionnaire, the Pittsburgh Sleep Quality Index (PSQI), and the Epworth Sleepiness Scale (ESS) [59–62]. The PSQI can assess sleep quality and disturbance with items that evaluate various aspects of sleep, and the ESS can measure the severity of daytime sleepiness. We will use the total scores as outcomes for each sleep measurement.

#### Constipation

We will assess the condition of constipation with the constipation assessment scale [63]. This scale is a self-report questionnaire consisting of 8 items. The total score represents the severity of constipation.

#### Obesity

We will calculate body mass index (BMI) (kg/m^2^) to evaluate obesity.

#### Mental health

The Profile of Mood States 2nd edition (POMS 2) will be used to evaluate a wide range of mood states [64, 65]. We will use the total mood disturbance score and 7 subscales (e.g., depression and tension–anxiety) as indicators of mental health status.

#### Physical and psychological symptoms

We will use the Anti-aging Quality of Life Common Questionnaire (AAQOL) to evaluate participants' health status, including physiological and psychological symptoms [66]. We will use the total score and subtotal scores for physical and psychological symptoms as indicators of an individual’s health status.

#### Health-related quality of life

We will use the self-reported MOS 36-item short-form health survey version 2 (SF-36v2) to evaluate health-related quality of life (HRQOL) [67, 68]. The physical and mental component summary scores will be used as indicators of HRQOL.

#### Daily health checks

We will ask participants to report their health status and body temperature on a daily check sheet. Specifically, participants will be asked to report the presence or absence of the following symptoms: sore throat, running nose, cough, phlegm, stomachache, diarrhea, loss of appetite, and lethargy/fatigue.

#### Habitual diet

We will administer the BDHQ both before and after the intervention, allowing us to assess baseline dietary intake as well as changes during the intervention period, including the intake of brown rice and mixed grains [31]. If there are significant between-group differences in the habitual intake of whole grains (including brown rice) at baseline between intervention groups or if significant changes in the habitual diet are observed during the intervention, these variables will be included as covariates in the statistical analyses.

All copyrighted questionnaires and tests to be used in this study will be obtained and administered in accordance with the relevant licensing and permission requirements.

### Plans to promote participant retention and complete follow-up {18b}

We will try to improve adherence to the intervention by asking participants to report their consumption of the supplement on the application and by sending a reminder message to participants each day if they do not report consumption by 19:00. Furthermore, we will provide additional incentives to participants for retention in the study if the data collected for endpoint evaluation are complete.

### Data management {19}

Blood and stool samples will be preserved properly after preprocessing for analysis. All cognitive assessments will be conducted using digital software on tablets and laptops to avoid errors associated with data entry. All data collected using paper-and-pen methods will be entered into spreadsheets and double-checked. Paper forms will be stored securely in a locked cabinet that only study staff will be able to access. All the research staff who complete the data collection will be trained to avoid missing data and will be asked to record their data collection processes for reference and to manage any potential data collection issues. For quality control, all data, regardless of collection methods, will be reviewed to identify response patterns, response ranges, out-of-range codes, and internal inconsistencies.

### Confidentiality {27}

All data and samples obtained in the trial will be stored securely at the study site in pseudonymized form, thus preventing participants from being identified without the corresponding table. The corresponding table will be stored electronically, and access will be limited to study staff members who handle inclusion and clinical data acquisition. All documents containing personal information, such as the informed consent form, will be stored separately from the study records. The data and samples will be archived for at least five years after the end of the study.

### Plans for collection, laboratory evaluation and storage of biological specimens for genetic or molecular analysis in this trial/future use {33}

Blood samples will be collected from 9:0012:00 after a 13-h fasting period. After collection, the blood samples will be divided to obtain PBMCs, and the plasma will be stored in a microtube at − 80 °C. For analyses of immune function and metabolism, total RNA will be extracted from PBMCs, after which RNA-seq libraries will be generated using the NEBNext Globin & rRNA Depletion Kit and the NEBNext UltraII Directional RNA Library Prep Kit. Sequencing experiments will be performed with the NextSeq 500 system. Data analysis will be performed as described in the “ Immune function” section.

Metabolites from plasma will be extracted using Bligh and Dyer’s method with some modifications. Hydrophilic metabolites will be analyzed will be performed using LC with a Discovery HS F5 column coupled with an LCMS-8060NX triple quadrupole mass spectrometer (Shimadzu, Kyoto, Japan) in MRM mode. Data analysis will be performed as described in the “ Metabolism” section.

At each visit, a stool sample will be collected with a kit containing a preservation solution and sent to the laboratory (TechnoSuruga Laboratory Co., Ltd., Shizuoka, Japan) to analyze the gut microbiota and SCFAs. The samples will be preprocessed for analysis immediately after arrival at the laboratory. Furthermore, microbial genomic DNA will be extracted from the stool samples, and template DNA will be subjected to 16S RNA gene amplicon sequencing using Illumina MiSeq.

## Statistical methods

### Statistical methods for primary and secondary outcomes {20a}

The co-primary outcomes will be analyzed to determine whether the consumption of the brown rice-derived supplement is superior to the use of a placebo in terms of positive health effects. The data derived from transcriptome and metabolome analysis will be compared between intervention groups after relevant covariates are controlled for. Microbiome and overall community composition will be compared between intervention groups on the basis of alpha and beta diversity with relevant covariates controlled for. For cognitive function, all outcomes will be compared through an analysis of covariance, with the intervention group set as a between-group factor, the data collection time points as a within-group factor, and the covariates as relevant covariates. The BMI and questionnaire data will be compared through an analysis of covariance controlling for relevant covariates. For continuous variables, the distribution of the data and the fulfillment of the model assumptions will first be assessed. Parametric methods will be used when the relevant assumptions are met; otherwise, appropriate nonparametric methods will be applied.

### Interim analyses {21b}

Not applicable, as no interim analyses are planned in this study.

### Methods for additional analyses (e.g., subgroup analyses) {20b}

Not applicable as, no additional analyses are planned in this study.

### Methods in analysis to handle protocol non-adherence and any statistical methods to handle missing data {20c}

The primary analysis will be based on the intention-to-treat (ITT) principle, but we will include only the randomized participants who participate in data collection at both the baseline and the study endpoint, irrespective of their degree of adherence to experimental food consumption. In this study, we will exclude randomized participants who drop out of the study and do not provide endpoint data, as this study aims to examine the effect of the nutritional supplement on healthy adults, and we do not expect as great an effect as when medication is administered to a patient group. To follow the ITT principle as closely as possible, all the participants will be asked to participate at the endpoint as much as possible. A per-protocol analysis limited to participants with adherence ratios greater than 80% will be performed as a sensitivity analysis. Every effort will be made to prevent missing values during the data collection process, and we will address any missing data using multiple imputation or regression imputation.

### Plans to give access to the full protocol, participant-level data and statistical code {31c}

After the termination of the study and publication of the results, access to the anonymized participant-level dataset will be provided upon reasonable request.

## Oversight and monitoring

### Composition of the coordinating center and trial steering committee {5d}

Regular monitoring of this study will be conducted by both the research team and the Ethics Committee of Tohoku University Graduate School of Medicine. Given that the study has a low-risk nature, the establishment of a monitoring committee and a trial steering committee was determined to be unnecessary. The research group comprises a principal investigator, a study coordinator, two physicians, two psychologists, two biologists, a biostatistician, a nurse, and a research assistant. The team will convene monthly to assess ongoing trial progress comprehensively. These meetings will include a review of study advancements, recruitment, data quality, retention rate, protocol amendments, and general research matters. In addition, the research team annually submits a comprehensive monitoring report to the Ethics Committee of Tohoku University Graduate School of Medicine.

### Composition of the data monitoring committee, its role and reporting structure {21a}

A data monitoring committee is not applicable because the present trial aims to examine the efficacy of consuming a health food that has been confirmed to be safe and pose a low risk to health.

### Adverse event reporting and harms {22}

Participants will be asked to actively report any adverse events that occur during the intervention period. The severity of any adverse events, irrespective of their relationship to the study intervention, will be documented in a case report form and assessed using the National Cancer Institute Common Terminology Criteria for Adverse Events v4.0.

### Frequency and plans for auditing trial conduct {23}

This study will be conducted under the auditing of the Ethics Committee of Tohoku University Graduate School of Medicine. The committee will oversee the quality and validity of the work and the researchers’ adherence to ethical standards throughout the study period through the annual report submitted by the study team. This process of auditing is independent of the investigators.

### Plans for communicating important protocol amendments to relevant parties (e.g., trial participants, ethical committees) {25}

Protocol amendments will be communicated to and approved by the Ethics Committee of Tohoku University Graduate School of Medicine according to standard policies and procedures, and updates to the UMIN-CTR will be made accordingly.

### Dissemination plans {31a}

We will present the results at national and international conferences and publish them in peer-reviewed journals. After all the results are published, the anonymized trial data will be available upon reasonable request. In addition, we plan to issue press releases to the interested public about the results of this trial.

## Discussion

Although brown rice is well recognized as a food that has various positive effects on human health, the underlying mechanisms of these effects in humans, especially in healthy individuals, remain unclear. This is the first study to provide insight into the effectiveness of consuming a brown rice-derived supplement on various aspects of health, including immune, cognitive, physical and mental health. Furthermore, we hypothesize that changes in the gut microbiota play a key role in these health effects; therefore, we will investigate how changes in the gut microbiota caused by the consumption of the brown rice-derived supplement affect health status. In addition, we aim to elucidate the mechanisms underlying the positive effects on health by performing omics analysis of blood samples. In contrast to previous human studies [13, 14, 69], it is very important from a public health perspective that this study focuses on the role of the brown rice-derived supplements in maintaining health among healthy individuals. The anticipated findings may have practical implications for health maintenance and prevention of lifestyle-related conditions in generally healthy adults. If the brown rice-derived supplement has favorable effects on immune and metabolic profiles and related health outcomes, it may represent a feasible diet-based option to support healthy aging. As the intervention is derived from a widely consumed staple food (rice), it may be acceptable and easy to incorporate into daily routines. We hope that the information derived from this study will be useful for promoting the use of brown rice-derived supplements as a health food from a public health perspective.

### Trial status

Recruitment has been ongoing since February 2023. The study is anticipated to conclude in the winter of 2025.

## Data Availability

All study investigators will have access to the final trial dataset. There are no contractual agreements that limit such investigators' access.

## Abbreviations

16S rRNA: 16S ribosomal ribonucleic acid
AAQOL: Anti-Aging Quality of Life Common Questionnaire
BMI: Body mass index
CANTAB: Cambridge Neuropsychological Test Automated Battery
DNA: Deoxyribonucleic acid
ESS: Epworth Sleepiness Scale
GABA: Gamma-aminobutyric acid
HRQOL: Health-related quality of life
ITT: Intention to treat
IQ: Intelligence quotient
MRM: Multiple reaction monitoring
PBMCs: Peripheral blood mononuclear cells
PCR: Polymerase chain reaction
POMS2: Profile of Mood States 2nd Edition
PSQI: Pittsburgh Sleep Quality Index
QIIME2: Quantitative Insights into Microbial Ecology version 2
SCFAs: Short-chain fatty acids
SF-36: Medical Outcomes Study 36-Item Short-Form Health Survey
UMIN-CTR: University Hospital Medical Information Network Clinical Trials Registry
WCST: Wisconsin Card Sorting Test
